# The ORF2 glycoprotein of hepatitis E virus inhibits cellular NF-κB activity by blocking ubiquitination mediated proteasomal degradation of IκBα in human hepatoma cells

**DOI:** 10.1186/1471-2091-13-7

**Published:** 2012-05-16

**Authors:** Bhavna Varshney, Sunil K Lal

**Affiliations:** 1 Virology Group, International Centre for Genetic Engineering & Biotechnology, New Delhi 110067, India; 2 Translational Health Science and Technology Institute, 496, Udyog Vihar Phase III, Gurgaon 122016, India

## Abstract

**Background:**

Nuclear factor kappa B (NF-κB) is a key transcription factor that plays a crucial role in host survival during infection by pathogens. Therefore, it has been a priority of many pathogens to manipulate the cellular NF-κB activity in order to create a favorable environment for their survival inside the host.

**Results:**

We observed that heterologous expression of the open reading frame 2 (ORF2) protein in human hepatoma cells led to stabilization of the cellular I kappa B alpha (IκBα) pool, with a concomitant reduction in the nuclear localization of the p65 subunit of NF-κB and inhibition of NF-κB activity. Although basal or TPA induced phosphorylation of IκBα was not altered, its ubiquitination was markedly reduced in ORF2 expressing cells. Further analysis revealed that ORF2 protein could directly associate with the F-box protein, beta transducin repeat containing protein (βTRCP) and ORF2 over expression resulted in reduced association of IκBα with the SKP1 and CUL1 components of the SCF^βTRCP^ complex. Chromatin immunoprecipitation (ChIP) assay of the proximal promoter regions of MHC-I heavy chain and IL-8 genes using p65 antibody and LPS stimulated ORF2 expressing cell extract revealed decreased association of p65 with the above regions, indicating that ORF2 inhibited p65 binding at endogenous promoters.

**Conclusions:**

In this report we suggest a mechanism by which ORF2 protein of HEV may inhibit host cell NF-κB activity during the course of a viral infection.

## Background

Nuclear factor kappa B (NF-κB) is a crucial transcription factor regulating multiple cellular pathways leading to survival or death of the cell depending on the stimulus. In unstimulated cells, the NF-κB dimers (p50/p65 heterodimer or p50/p50 homodimer) are retained in the cytoplasm in an inactive form as a consequence of their association with members of another family of proteins called I kappa B (IκB). Upon stimulation by activators like tumor necrosis factor alpha (TNF-α), interleukin 1 (IL-1), CD40L, lipopolysaccharides (LPS) etc., signaling cascades involving activation of various protein kinases are initiated that result in the recruitment and activation of the IκB kinases (IKKs) which phosphorylate IκBα, leading to its degradation by the 26S proteasome complex. The degradation of IκBα exposes the p50/p65 nuclear localization sequence and allows NF-κB dimers to translocate to the nucleus, bind to κB motifs in the promoters regions of many genes, and regulate their transcription [1].

In many cases, infection by extra-cellular pathogens has been shown to alter NF-κB activity in order to facilitate the survival of pathogens or host. As part of the host defense mechanism against invading pathogens, NF-κB activation is required for resistance to a variety of viral, bacterial, and parasitic infections [1]. However, many viruses such as HIV, exploit this property to their benefit by driving their gene expression through κB response elements located in their promoters [2]. On the contrary, many pathogens such as African swine fever virus, HIV-1 and cowpox virus [3-5] have developed strategies to interfere with host NF-κB responses. Inhibition of NF-κB activity by these pathogens has been shown to be important for pathogenesis.

Hepatitis E virus (HEV) is a positive strand RNA virus which codes for three known open reading frames (ORFs) [6]. ORF1 codes for non structural proteins, essential for viral replication; ORF2 codes for the major capsid protein of HEV, called ORF2 protein; and ORF3 codes for a phosphoprotein which may play a key role in manipulating various host-cell processes during viral infection, and may have a role in cell survival and propagation of the virus [7,8]. Although HEV infection is generally self-limiting, it induces fulminant hepatic failure, which results in a very high mortality rate in pregnant women. A recent study done by Prusty and coworkers has demonstrated that NF-κB activity is suppressed in the PBMC and liver biopsy samples of pregnant fulminant hepatic failure patients [9]. However, the mechanism underlying this phenomenon remains unknown.

In the present study, we report the ability of the ORF2 protein to inhibit the cellular NF-κB activity. In human hepatoma cells, ORF2 protein could directly associate with the F-box protein βTRCP and heterologous expression of the ORF2 protein led to reduced recruitment of SKP1 and CUL1 subunits to the SCF^βTRCP^ ubiquitination complex, resulting in decreased ubiquitination and degradation of the IκBα protein. This, in turn, led to reduced nuclear localization and subsequent DNA binding of the p65 protein, which is the major subunit of the NF-κB trans-activation complex. Analysis of two NF-κB target genes further confirmed the above observation. The possible significance of this phenomenon in enhancing survival of HEV infected hepatocytes is discussed.

## Results

### Heterologous expression of the ORF2 protein inhibits NF-ÎºB activity

**Figure 1:**
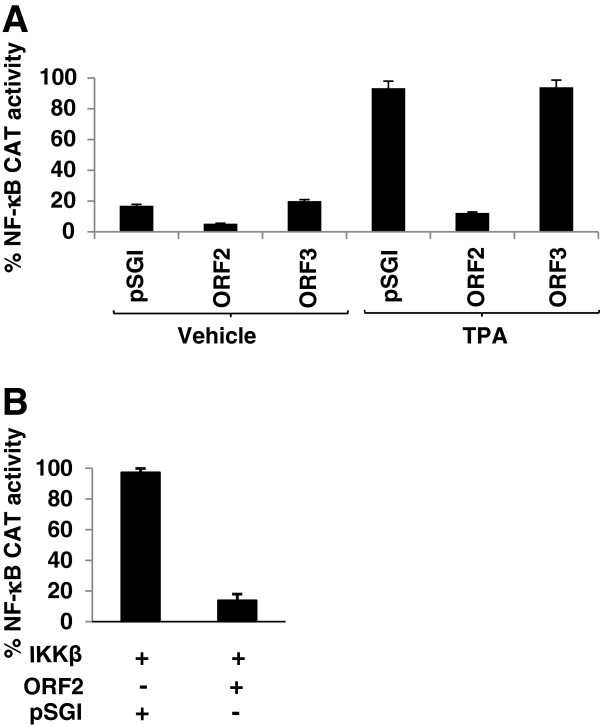
**Heterologous expression of ORF2 protein inhibits NF-κB activity.** (**A**) Huh7 cells were cotransfected in duplicate with NF-κB CAT reporter and pSGI vector or pSGI-ORF2 expression plasmid. 48 hour post transfection, cells were treated with 100 ng/ml TPA for 30 minutes and CAT activity was assayed. Spot intensity was quantified using NIH Image program and percentage CAT activity was calculated assuming the highest value as 100%. Graph represents mean ± SEM of 3 independent sets of experiments. (**B**) Huh7 cells were cotransfected in duplicate with NF-κB CAT reporter, IKKβ expression plasmid and pSGI vector or pSGI ORF2 expression plasmid. 48 hour post transfection, CAT activity was assayed. Graph represents mean ± SEM of 3 independent sets of experiments.

In order to test whether ORF2 or ORF3 protein of HEV inhibit cellular NF-κB activity, a reporter vector with IL-2 receptor promoter region, which contains NF-κB element, cloned upstream of the chloramphenicol acetyl transferase coding sequence (NF-κB CAT; [10] was used. This vector was inducible by NF-κB activating agents like TPA or IL-1β. Huh7 cells were transiently transfected with the NF-κB CAT vector along with either ORF2 or ORF3 expression plasmids. Assay of chloramphenicol acetyl transferase (CAT) activity using these cell extracts revealed that ORF2 protein inhibited the NF-κB CAT activity (Figure 1A). However, no inhibition was observed by ORF3 expression (Figure 1A). In order to investigate whether ORF2 mediated inhibition of NF-κB activity was an artifact of the experimental system, cells were treated for 30 minutes with Phorbol 12-myristate 13-acetate (TPA), a known inducer of NF-κB activity [11], or transfected with an expression construct of IκB kinase β, which is the catalytic subunit of the IKK complex that acts as a constitutively active inducer of NF-κB activity [12]. TPA treatment increased NF-κB activity of mock transfected cells by approximately 4 folds whereas ORF2 expressing cells did not show any significant increase in NF-κB activity (Figure 1A). Similarly, co-expression of ORF2 in IKKβ transfected cells resulted in downregulation of NF-κB activity in comparison to cells transfected with only IKKβ (Figure 1B). These results clearly suggest that HEV ORF2 inhibits NF-κB activity.

**Figure 2:**
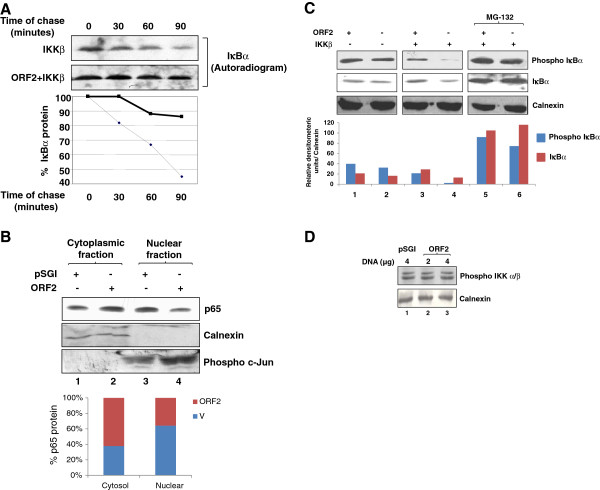
**ORF2 protein increases the half life of cellular IκBα protein.** (**A**) Huh7 cells were cotransfected with IKKβ and pSGI or pSGI ORF2 expression plasmids and pulse chased, as described in methods. Total IκBα was immunoprecipitated and samples were resolved on 12% SDS-PAGE and bands were detected by autoradiography. Band intensities were quantified using NIH Image program and graph was plotted assuming protein level at zero time to be 100%. In the graph, thin dotted line represents IKKβ transfected sample and solid line represents ORF2 and IKKβ cotransfected samples. Similar results have been obtained in 3 independent experiments. (**B**) Cytoplasmic (lane 1, 2) and nuclear (lane 3, 4) fractions of pSGI or pSGI ORF2 transfected cells were immunoprecipitated and subsequently western blotted with anti-p65 antibody (upper panel). A fraction of lysates were western blotted with calnexin and phospho c-Jun antibodies. Graph represents quantitation of p65 band intensities from the above image. Similar results have been obtained in 2 independent experiments. (**C**) Huh7 cells were transfected with expression plasmids: Lane 1, ORF2; lane 2, pSGI vector; lane 4 and 6, IKKβ; lane 3 and 5, ORF2 and IKKβ. Cell lysates were immunoblotted with phospho-IκBα, (ser 32), IκBα and calnexin antibodies. MG-132 was added 3 hours prior to cells harvesting. Graph shows quantitative representation of calnexin value normalized band intensities of phospho- IκBα and total IκBα from the above image. Similar results have been obtained in 3 independent experiments. (**D**) pSGI vector or pSGI ORF2 (lane 2 and 3) transfected cell lysates were immunoblotted with phospho IKKα/β (ser 180/181) and calnexin antibodies. 2 μg & 4 μg denotes the respective amount of DNA transfectedh.

Further, we examined the turnover of IκBα in IΚKβ and ORF2 co-expressing cells since IκBα is the major inhibitor of NF-κB activity in unstimulated cells. By performing a pulse chase assay, using ^35^S]-cys/met labeling mix, the half-life of IκBα in IKKβ expressing cells was found to be approximately 90 minutes, whereas in cells expressing both IKKβ and ORF2, approximately 15% of IκBα was found to be degraded at a chase period of 90 minutes (Figure 2A). Notably, the levels of IκBα at the end of the labeling period (0 minute chase) in IKKβ alone expressing cells were less than that in IKKβ and ORF2 coexpressing cells, indicating that IκBα is a very labile protein in IKKβ expressing cells. The graph represents quantitative measurement of band intensities of IκBα at the indicated time points assuming the band intensity at the onset of the chase as 100%. To further confirm whether IκBα stabilization actually blocked nuclear translocation of NF-κB in ORF2 expressing cells, nuclear and cytoplasmic fractions were separated in mock and ORF2 transfected cells and equal amounts of samples were immunoprecipitated with anti-p65 antibody. As seen in Figure 2B, ORF2 transfected cells were found to accumulate more p65 protein in the cytoplasmic fraction than in the nuclear fraction (Figure 2B, lane 2 and 4; see the densitometric estimation of band intensities in the graph below). As expected, mock transfected cells were found to contain more p65 protein in the nuclear fraction (Figure 2B, lane 1 and 3). This is attributed to the fact that basal NF-κB activity is higher in Huh7 cells [13]. In order to check the purity of fractions obtained in nuclear and cytoplasmic fractions and as loading controls, equal amounts of both fractions from each sample were immunoblotted with anti-calnexin and anti-phospho c-Jun antibodies.

In order to check whether the phosphorylation of IκBα was altered in ORF2 expressing cells, the level of phosphorylated IκBα (ser32) was measured in ORF2 expressing cells. ORF2 expression marginally increased the levels of phospho IκBα (Figure 2C, upper panel, lane 1 and 2; see the densitometric estimation of band intensities in the graph below) as well as the level of total IκBα (middle panel, lane 1 and 2). In IKKβ and ORF2 co-expressing cells, the level of total as well as phosphorylated IκBα was significantly higher as compared to only IKKβ expressing cells (Figure 2C, lane 3 and 4). In order to check that the decreased band intensity in IKKβ expressing cells was due to accelerated degradation of IκBα, one set of cells were also treated with the proteasome inhibitor MG-132 for 2 hours, resulting in equal IκBα protein levels in both the samples (Figure 2C, lane 5 and 6). Aliquots of the sample were immunoblotted with anti-calnexin antibody to check equal loading. Thus, it appears that ORF2 expression prevents the degradation of IκBα. The fact that ORF2 did not interfere with phosphorylation of IκBα by the IKK complex was further confirmed by checking the activity of the IKK complex. ORF2 expressing cell lysate was immunoblotted for phospho IKKα/β (ser180/181) levels. As expected, ORF2 expression (2 or 4 μg plasmid DNA transfection) did not modulate the levels of pIKK α/β (Figure 2D).

### ORF2 protein interferes with IÎºBÎ± ubiquitination

**Figure 3:**
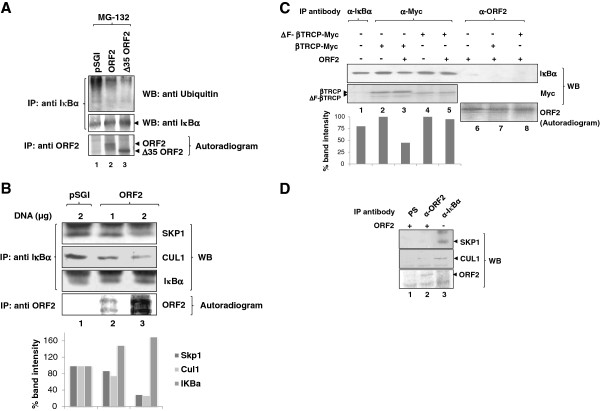
**ORF2 protein interferes with IκBα ubiquitination.** (**A**) Huh7 cells transfected with pSGI vector, ORF2 (full-length) and Δ35 ORF2 expression constructs were labeled metabolically with [^35^S]-cys/met promix and treated with MG-132 for 2 hours prior to lysis. Lysates were immunoprecipitated with anti-IκBα antibody and immunoblotted with anti-ubiquitin antibody (upper panel). Same blot was stripped and reprobed with IκBα antibody (middle panel). An aliquot of the lysate was immunoprecipitated with anti-ORF2 antibody and radioactive bands were detected by autoradiography (lower panel). (**B**) Huh7 cells transfected with pSGI (lane 1) or ORF2 (increasing DNA amounts, lane 2 and 3) expression plasmids were metabolically labeled with [^35^S]-cys/met promix. Lysates were immunoprecipitated with IκBα antibody and immunoblotted with SKP1, CUL1 and IκBα antibodies. An aliquot of lysate was immunoprecipitated with anti-ORF2 antibody and radioactive bands were detected by autoradiography. Graph represents percent band intensities of Skp1, Cul1 and IκBα (from lanes 1–3) levels, considering the intensity of pSGI transfected band in lane 1 as 100%. Similar results have been obtained in 3 independent experiments. (**C**) Huh7 cells expressing the indicated protein were immunoprecipitated with the respective antibodies and immunoblotted anti-IκBα antibody (upper panel). Same blot was stripped, cut into halves and immunoblotted with anti-myc (lower pane, lane 1–5) or anti-ORF2 (lane 6–8) antibodies. In lane 1, 50% of the sample has been loaded in comparison to other lanes. Graph represents percent IκBα band intensity, considering the intensity of IκBα band in lane 2 as 100%. Similar results have been obtained in 3 independent experiments. (**D**) pSGI vector (lane 3) or pSGI ORF2 (lane 1 and 2) transfected cells were labeled with [^35^S]-cys/met promix and lysates were immunoprecipitated with anti-IκBα antibody, pre-immune serum (PS) or anti-ORF2 antibodies, respectively; subsequently, samples were immunoblotted with SKP1 and CUL1 antibodies. Same blot was air dried and exposed to X-ray film to check for ORF2 expression by autoradiography.

Proteasomal degradation of IκBα is preceded by its ubiquitination, which occurs by the association of phosphorylated IκBα with the SCF^βTRCP^ complex [14]. In order to check whether ORF2 inhibits IκBα ubiquitination, we checked the level of ubiquitinated IκBα in ORF2 expressing cells. Mock or ORF2 transfected cells were treated with MG-132 for 2 hours, IκBα was immunoprecipitated, followed by immunblotting with anti-ubiquitin antibody. Protein level of ubiquitinated IκBα was significantly decreased in full-length ORF2 expressing cells as compared to control cells (Figure 3A top panel, lane 1 and 2). A similar effect (lane 3) was observed following expression of a mutant ORF2 protein (Δ35) with ER signal sequence deleted which consequently constitutively localizes to the cytoplasm [15]. Aliquots of the immunoprecipitated lysate were immunoblotted with IκBα antibody to confirm its presence (Figure 3A, middle panel). A parallel set of samples were labeled with ^35^S]-cys/met promix, immunoprecipitated with anti-ORF2 antibody and autoradiographed to check the expression of full-length and Δ35 ORF2 protein (Figure 3A, bottom panel).

Subsequently, we checked whether ORF2 interfered with the assembly of IκBα ubiquitination machinery. Expression of ORF2 protein inhibited the association of IκBα with SKP1 (Figure 3B, top panel) and CUL1 (Figure 3B, 2^nd^ panel) in a dose-dependent manner (compare lane 1 with lanes 2 and 3). Aliquots of the sample were immunoblotted with anti-IκBα antibody to check the levels of IκBα (3^rd^ panel). A parallel set of samples were labeled with [^35^S]-cys/met and immunoprecipitated with anti-ORF2 antibody to check the expression of ORF2 (Figure 3B, 4^th^ panel). To further check whether the ORF2 expression in these cells inhibited the association of IκBα with the F-box protein βTRCP, ORF2 and myc tagged βTRCP co-expressing cells were labeled with [^35^S]-cys/met and aliquots of the lysate were immunoprecipitated with anti-myc antibody and immunoblotted using anti-IκBα antibody. ORF2 expression led to the inhibition of IκBα association with full-length βTRCP when compared to control cells (Figure 3C, lane 3 and 2, respectively). However, IκBα association with an F-box deleted mutant βTRCP (ΔF-βTRCP) remains unaffected despite the presence of ORF2 (Figure 3C, lane 5). The same blot was stripped and reprobed with anti-myc antibody to check the expression of full-length and ΔF-βTRCP (Figure 3C, lower panel, lane 1–5). Lane 1 shows the protein level of total IκBα. 50% of the sample has been loaded in lane 1 in comparison to other samples. The other half of the blot was autoradiographed to check the expression of the ORF2 protein (Figure 3C, lower panel, lane 6–8).

Since the presence of ORF2 could inhibit IκBα association with full-length βTRCP but not with ΔF-βTRCP, we postulated that the ORF2 protein either interacts with IκBα and sequesters it away from βTRCP or interacts with other subunits of the SCF complex thereby modulating βTRCP binding to IκBα. Alternatively, it binds to βTRCP itself and blocks the formation of the SCF complex. We thus checked the association of ORF2 with the above components. No interaction was detected between ORF2 and IκBα (Figure 3C, lane 6) as judged by coimmunoprecipitation assay. The possibility that βTRCP over-expression might be promoting ORF2 association with IκBα was ruled out by testing ORF2 and IκBα association in full-length or ΔF- βTRCP over-expressing cells (Figure 3D, lane 7 and 8). Lane 1 shows the level of IκBα in control cells. Subsequently, we checked whether ORF2 interacts with SKP1 and CUL1. The ORF2 protein did not associate with either SKP1 or CUL1 (Figure 3D, lane 1 and 2) under conditions, where IKBα associated with these proteins (lane 3).

### ORF2 protein directly associates with the F-box protein Î²TRCP

**Figure 4:**
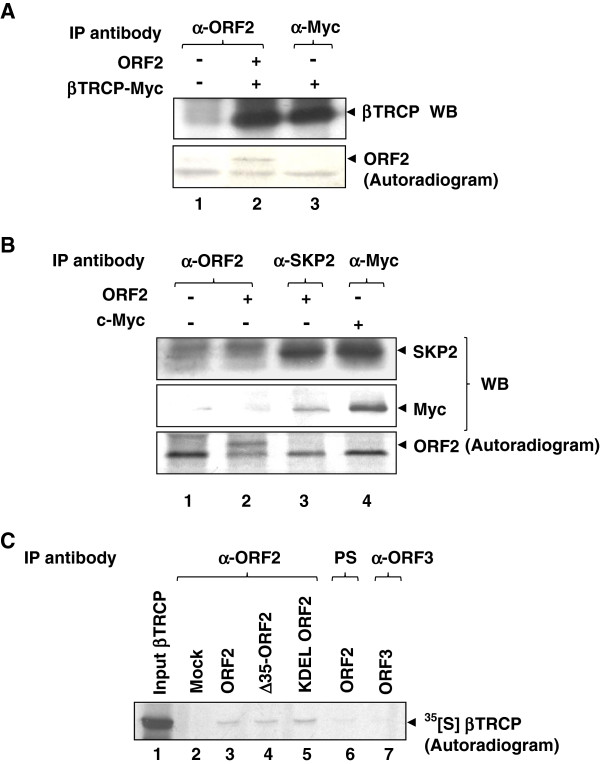
**ORF2 protein associates with βTRCP protein.** (**A**) Huh7 cells were transfected with pSGI, ORF2 and myc tagged βTRCP expression constructs in indicated combinations and labeled with [^35^S]-cys/met promix. Lysates were immunoprecipitated with anti-ORF2 (lane 1 and 2) or anti-Myc (lane 3) antibody and immunoblotted with anti-Myc antibodies. Immunoblot presented in upper panel was air dried and exposed to X-ray film to check for ORF2 expression (lower panel). (**B**) Huh7 cells transfected with pSGI vector or ORF2 or c-Myc expression constructs were labeled with [^35^S]-cys/met promix, immunoprecipitated with the indicated antibodies and immunoblotted with anti-SKP2 or anti-Myc antibodies. Myc blot was air dried and exposed to X-ray film to check for ORF2 expression (3^rd^ panel). (**C**) Lysates from mock (lane 2), ORF2 (lane 3 and 6), Δ35 ORF2 (lane 4) and KDEL ORF2 (lane 5) transfected Huh7 cells, were immunoprecipitated using anti-ORF2 antibody (lane 2–5) or pre-immune serum (PS, lane 6) and incubated along with *in vitro* translated [^35^S]-cys/met labeled full-length βTRCP protein (lane 2–6). Bound βTRCP protein band was visualized by autoradiography. Lane 1 represents total βTRCP protein used in each sample. For negative control, ORF3 expressing cells were immunoprecipitated with anti-ORF3 antibody and processed as above (lane 7).

Coimmunoprecipitation assay of cell lysate expressing both ORF2 and myc tagged full-length βTRCP revealed that ORF2 could efficiently associate with the latter (Figure 4A, upper panel, lane 2). Exposure of the same blot to X-ray film revealed the band specific to ORF2 in βTRCP and ORF2 cotransfected cells (Figure 4A, lower panel, lane 2). 3^rd^ lane in upper panel shows the expression of the βTRCP protein, as judged by immunoprecipitation using anti-myc antibody. Specificity of the above interaction was further verified by checking for association of ORF2 with another F-box protein, SKP2. We were unable to detect any interaction between ORF2 and SKP2 as judged by coimmunoprecipitation assay (Figure 4B, top panel). In order to ensure that SKP2 was indeed functional in these experiments, the same blot was stripped and reprobed with anti-myc antibody. Endogenous SKP2 was able to coprecipitate endogenous as well as overexpressed c-myc protein (Figure 4B, middle panel) and similarly IP using myc antibody revealed its interaction with the SKP2 protein (Figure 4B, lane 4, upper and middle panels). Bottom panel shows an autoradiogram of ORF2 expression. These experiments proved that ORF2 specifically interacts with the F-box protein βTRCP. Direct interaction between ORF2 and βTRCP was further confirmed using an *in vitro* pull-down assay. Full-length ORF2 as well as KDEL ORF2 (ORF2 protein containing “KDEL” amino acids at its C-terminal end, which allows it to be retained in the endoplasmic reticulum instead of getting transported through the golgi) and Δ35 ORF2 mutant were able to pull-down βTRCP (Figure 4C, lane 3, 4, 5). As negative controls, ORF2 transfected sample was immunoprecipitated with pre-immune serum (Figure 4C, lane 6) or mock translated lysate was incubated with ORF2 protein bound to beads (Figure 4C, lane 2) or, the ORF3 protein was incubated with βTRCP protein (Figure 4C, lane 7).

### The ORF2 protein downregulates the expression of NF-ÎºB targets

**Figure 5:**
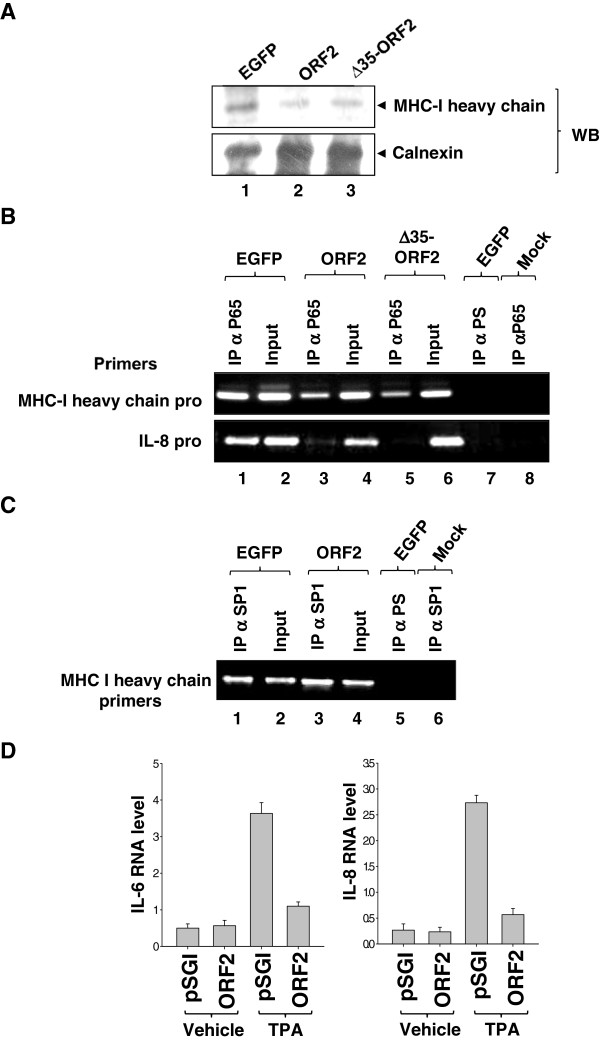
**ORF2 protein inhibits the expression of MHC-I heavy chain.** (**A**) EGFP, ORF2 or Δ35 ORF2 expressing cells were treated with LPS for 45 minutes after 48 hours of transfection. Lysates were immunoprecipitated and immunoblotted with anti-MHC-I heavy chain antibody (upper panel). An aliquot of the lysate was immunoblotted with anti-calnexin antibody (lower panel). (**B**) To test p65 NF-κB binding to the MHC-I heavy chain and IL-8 promoters ChIP assay was performed. Huh7 Cells were transfected with EGFP, ORF2 or Δ35 ORF2 expression plasmids and treated with LPS for 45 minutes prior to harvesting. PCR was performed using primers flanking the proximal promoter region of MHC-I heavy chain and IL-8 genes. Lane 7 shows ChIP from EGFP expressing cells immunoprecipitated with rabbit pre-immune serum. Lane 8 shows ChIP from mock immuonprecipitation (without cell lysate) using p65 antibody. 10% of the initial amount of chromatin used for one immuonprecipitation assay was used as input in each PCR. (**C**) SP1 binding to the MHC-I heavy chain ptomoter was again assayed by ChIP. Huh7 Cells were transfected with EGFP or full-length ORF2 expression plasmids and treated with LPS for 45 minutes prior to harvesting. PCR for MHC-I promoter regions was performed using primers mentioned above. Lane 5 shows ChIP from EGFP expressing cells immunoprecipitated with rabbit pre-immune serum. Lane 6 shows ChIP from mock immunoprecipitation using SP1 antibody. 10% of the initial amount of chromatin used for one immuonprecipitation assay was used as input in each PCR. (**D**) Quantitative RT-PCR analysis of IL-6 and IL-8 RNA level in Huh7 cells, treated as indicated. Values were normalized with respect to that of HPRT and represented as mean ± SEM.

NF-κB is known to regulate the transcription of a large number of genes during different cellular conditions. One of the most critical events for initiation of immune response against viral antigens is the presentation of viral peptide epitopes over the infected cell surface, which can then be recognized by cytolytic T cells. The viral antigens are presented through their association with the major histocompatibility complex-I (MHC-I). As MHC-I heavy chain is a known target of NF-κB, we checked expression levels of the MHC-I heavy chain in ORF2 expressing cells stimulated with bacterial lipopolysacharides (LPS). EGFP (Figure 5A, lane 1), full-length ORF2 (Figure 5A, lane 2) or Δ35-ORF2 (Figure 5A, lane 3) transfected cells were treated with LPS for 45 minutes and total cell lysate was immunoprecipitated and immunoblotted with anti-MHC-I heavy chain antibody (Figure 5A, upper panel). Protein level of MHC-I heavy chain was decreased in both full-length and Δ35-ORF2 expressing cells in comparison to EGFP expressing cells. An aliquot of the lysate was immunoblotted with anti-calnexin antibody to ensure equal loading of the sample (lower panel).

Further, we checked NF-κB recruitment to the MHC I heavy chain promoter in LPS stimulated ORF2 expressing cells by chromatin immunoprecipitation (ChIP) assay. Immunoprecipitation was conducted using an antibody specific for the p65 subunit of the NF-κB complex. EGFP expression did not alter p65 recruitment to MHC-I heavy chain promoter. However, full-length or Δ35-ORF2 expression decreased p65 recruitment to the MHC-I heavy chain promoter. 10% of the total lysate used for one immuonprecipitation reaction was used as input in each sample (Figure 5B, upper panel). We also checked p65 recruitment to interleukin 8 (IL-8) proximal promoter region, which showed a similar pattern as observed for the MHC-I heavy chain promoter (Figure 5B, lower panel). As a control to check whether the observed phenomenon was specific for NF-κB, aliquots of the LPS treated lysate were immunoprecipitated with anti-SP1 antibody (specific for SP1 transcription factor, which also binds to MHC-I heavy chain promoter) and purified ChIP DNA was PCR amplified using MHC-I heavy chain promoter specific primer. As expected, SP1 recruitment to the MHC-I heavy chain promoter was not altered in ORF2 expressing cells (Figure 5C). These experiments confirmed that ORF2 expression specifically prevents p65 NF-κB association with its cognate response element present on natural promoters.

Next, the effect of ORF2 protein on the expression of two TPA inducible cytokines- IL-6 and IL-8 was measured by performing real time quantitative RT-PCR of these cytokine transcripts in ORF2 expressing Huh7 cells, which were treated with TPA for 6 hours prior to RNA isolation. As expected, IL-6 and IL-8 transcript level was decreased in

ORF2 expressing TPA treated cells in comparison to mock transfcetd TPA treated cells (Figure 5D). These experiments indicates that ORF2 protein, by virtue of its ability to inhibit NF-κB activity, suppress TPA induced IL-6 and IL-8 RNA synthesis.

## Discussion

The ORF2 protein of HEV has traditionally been believed to associate with genomic RNA, multimerize and form the viral capsid. No other function of ORF2 protein has yet been reported. In this article, we present evidence which suggest that the ORF2 protein may be playing an important regulatory role during the viral life cycle. The fact that the observed phenomenon was not an artifact of the experimental setup was evident from multiple experiments. First, ORF2 was capable of directly interacting with the F-box protein βTRCP, both *in vitro* and in vivo. Second, Δ35 ORF2 mutant that is unable to translocate to the endoplasmic reticulum (ER), was also capable of inhibiting NF-κB activity, thus ruling out the possibility of ER stress induced artifact. Third, ORF3 protein of HEV was unable to elicit such a function in parallel experiments.

HEV infection results in an acute, self-limiting and icteric disease that is prevalent in much of the developing world. Although self-limiting infection occurs in adults with mortality rate ~1–2%, a high 10–20% mortality rate is observed during pregnancy [6]. Hence it is important to understand the molecular mechanism by which HEV completes its life cycle inside the host. A recent study done by Prusty and coworkers revealed that NF-κB activity is inhibited in the PBMC and liver of fulminant hepatic failure patients [9]. Our findings that the ORF2 protein has the ability to inhibit NF-κB activity in human hepatoma cells provide a possible molecular explanation to their observation.

The NF-κB inhibitory activity of the ORF2 protein may be mediated by its ability to directly associate with the F-box protein βTRCP and inhibit the assembly of the IκBα ubiquitination complex. βTRCP is a cytoplasmic protein. Hence it is worth speculating that the ORF2 βTRCP interaction would occur in the cytoplasm. Although ORF2 is an N-linked glycoprotein, which is cotranslationally translocated to the endoplasmic reticulum, recent studies performed in our laboratory have demonstrated that a fraction of ORF2 protein exploits the ER retro-translocation machinery to get access to the cytoplasm, where it is detected as a non-glycosylated protein. Importantly, in contrast to other retrotranslocated substrates, retrotranslocated ORF2 protein is not immediately degraded by the 26S proteasome complex present in the cytoplasm [16]. Thus, cytoplasm localized deglycosylated ORF2 protein may be able to associate with βTRCP. Indeed, experiments done using Δ35 ORF2 protein suggest that non-glycosylated, cytoplasm localized ORF2 protein is capable of associating with βTRCP and inhibiting NF-κB activity. Further studies need to be undertaken to verify the exact mechanism.

Expression of MHC class I heavy chain, which is known to be a transcriptional target of NF-κB, was down-regulated in LPS treated ORF2 expressing cells presumably due to reduced promoter occupancy of p65, which is crucial for NF-κB dependent transcriptional activation. In an infected cell, by default, pathogenic antigens are processed by the proteasome and presented by the MHC class I molecules so that the former can be recognized by cytolytic T cells. This helps in pathogen clearance at an early stage of infection. Thus, it is beneficial for any pathogen to escape this step. In fact, downregulation of MHC class I both at transcriptional and post-translational level is observed in many pathogenic infections [17-19]. Since NF-κB is one of the major transcription factors induced during pathogen infection that enhances gene expression of many chemokines and class I molecules in the infected cell, inhibition of the NF-κB activity by pathogen encoded proteins will ensure evasion of host immune response at an early stage; thus providing the pathogen a time window to establish successful infection.

## Conclusions

Owing to its central role in regulating multiple cell signaling pathways, modulation of NFKB activity has been an attractive target of many viral factors in order to allow them to exploit the host cell signaling machinery to their benefit. We have identified the ability of the ORF2 protein of the HEV to inhibit host cell NFKB activity. However, whether such an event is recapitulated during the natural course of HEV infection and whether it is crucial for the virus induced pathogenic effects, can be answered only by performing similar experiments using a model organism infected with the wild type virus. Nevertheless, the present study confirms the NFKB inhibitory property of the HEV ORF2 protein and provides some evidence suggesting a plausible mechanism underlying this event.

## Methods

### Plasmids and reagents

Cloning of ORF2 and Δ35 ORF2 in pSGI has been described earlier [15]. Flag tagged IKKβ [12], IL-2 receptor α promoter containing NF-κB response element cloned upstream of the chloramphenicol acetyl ransferase (CAT) coding sequence (NF-κB CAT) [10], Wild-type, ΔF βTRCP cDNA in pCDNA3.1 [20] and pSGI c-myc [21] constructs were gifts from Drs. Rene Bernards, Ranjan Sen, Richard Benarous and Vijay Kumar, respectively. All DNA constructs used for mammalian cell transfection were purified by cesium chloride gradient centrifugation [22].

All antibodies were purchased from Santa Cruz Biotechnology Inc. (California, USA); TPA and MG-132 were purchased from Calbiochem Chemicals (California, USA). LPS was purchased from Sigma-Aldrich (USA). [^35^S]-cysteine/methionine labeling mix was obtained from New England Nuclear (Massachusetts, USA).

### Cell culture and transfection

Human Hepatoma (Huh7) cells were maintained in DMEM supplemented with penicillin, streptomycin and 10% fetal bovine serum. Cells were transfected with Lipofectin/Lipofectamine 2000 reagent (Invitrogen Corp., California, USA) as per manufacturer’s instructions. For negative control conditions, cells were transfected with their respective empty vectors. In all transfections, total DNA amount was equalized by adding pSGI vector.

### Metabolic labeling, immunoprecipitation and immunoblotting

Radiolabeling of cells with ^35^S]-cys/met promix, immunoprecipitation and immunoblotting was done as described earlier [8]. Data obtained is representative of at least 3 independent sets of experiments conducted. Results were quantified using the NIH Image V: 1.32 program, normalized values calculated and graphs plotted wherever indicated. All the inhibitors were added during starvation period and maintained throughout the labeling period.

Effective concentrations of different compounds used: TPA 100 ng/ml, MG-132 50 μM, LPS 10 μg/ml.

### Pulse chase assay

Cells were pulse-labeled for 20 minutes with 250 μCi of ^35^S]-cys/met labeling mix and chased in complete medium for the indicate time periods, followed by immunoprecipitation, as described before [23].

### Chloramphenicol acetyl transferase (CAT) assay

Forty eight hours post-transfection, cells were harvested in phosphate buffer saline and CAT assay was conducted as described elsewhere [21].

### *In vitro* protein expression

βTRCP protein was expressed *in-vitro* using pCDNA-βTRCP expression construct in a coupled transcription-translation reaction (TNT kit, Promega Corp., Wisconsin, USA) following manufacturers protocol.

### Nuclear fractionation

Nuclear fractionation was performed as described earlier [23].

### Chromatin immunoprecipitation (ChIP) assay

Cells cultured in 60 mm dish were transfected in triplicate with respective plasmids. Control samples were transfected with EGFP expression plasmid (pSGI-EGFP). Forty-eight hours post-transfection, cells were fixed in 1% formaldehyde and ChIP assay was performed as described elsewhere [24]. Forward and reverse primer sequences for amplification of MHC-I heavy chain promoter were 5^′^ CAGGGAGTCCAGTTCAGGGA 3^′^ and 5^′^ TGAGTCCGGGTGGGTGCGTG 3^′^, respectively and for amplification of IL-8 promoter were 5^′^ GGGCCATCAGTTGCAAA TC 3^′^ and 5^′^ TTCCTTCCGGTGGTTTCTTC 3^′^, respectively.

### RNA isolation and real time quantitative RT-PCR analysis

RNA was isolated from Huh7 cells using Trizol reagent (Invitrogen) following manufacturers protocol, followed by reverse transcription and real time quantitative RT-PCR analysis as described [25].

## Authors’ contributions

MS contributed towards all the molecular biology work, transfection work and CAT and pulse-chase assays and acquisition of data and data analysis and interpretation BV contributed to the RNA based work and ChIP assays and revised the manuscript critically SKL was involved in conception and design drafting initial experiments and later the manuscript. Each author has participated sufficiently in the work to take public responsibility for appropriate portions of the content. All authors read and approved the final manuscript.
